# To eat or not to eat mitochondria? How do host cells cope with mitophagy upon bacterial infection?

**DOI:** 10.1371/journal.ppat.1011471

**Published:** 2023-07-06

**Authors:** Jérémy Verbeke, Xavier De Bolle, Thierry Arnould

**Affiliations:** 1 Research Unit in Cell Biology, Laboratory of Biochemistry and Cell Biology URBC)–Namur Research Institute for Life Sciences (NARILIS), University of Namur, Namur, Belgium; 2 Research Unit in Microorganisms Biology (URBM)–Namur Research Institute for Life Sciences (NARILIS), University of Namur, Namur, Belgium; Washington University School of Medicine in Saint Louis: Washington University in St Louis School of Medicine, UNITED STATES

## Abstract

Mitochondria fulfil a plethora of cellular functions ranging from energy production to regulation of inflammation and cell death control. The fundamental role of mitochondria makes them a target of choice for invading pathogens, with either an intracellular or extracellular lifestyle. Indeed, the modulation of mitochondrial functions by several bacterial pathogens has been shown to be beneficial for bacterial survival inside their host. However, so far, relatively little is known about the importance of mitochondrial recycling and degradation pathways through mitophagy in the outcome (success or failure) of bacterial infection. On the one hand, mitophagy could be considered as a defensive response triggered by the host upon infection to maintain mitochondrial homeostasis. However, on the other hand, the pathogen itself may initiate the host mitophagy to escape from mitochondrial-mediated inflammation or antibacterial oxidative stress. In this review, we will discuss the diversity of various mechanisms of mitophagy in a general context, as well as what is currently known about the different bacterial pathogens that have developed strategies to manipulate the host mitophagy.

## 1. The many regulations of mitochondrial integrity: A story of biogenesis, quality control, and degradation

In the light of their unique and intricate evolution, mitochondria developed into complex, motile, and highly dynamic organelles providing a variety of functions and signalling hubs for eukaryotic cells [1]. Besides their critical role in efficient ATP production, mitochondria are also specialised in many other essential cellular functions such as lipid metabolism, calcium homeostasis, redox signalling, synthesis of [Fe-S] clusters, control of DNA epigenetics and chromatin remodelling, integration of programmed cell death signals, cell differentiation control, as well as regulation of the innate immunity and inflammation [2]. However, mitochondria are constantly exposed to several external and internal stresses, including nutrient deprivation or oxidative stress, which could lead to oxidative phosphorylation uncoupling, mitochondrial DNA damage, and/or impaired proteostasis [3]. Given the complexity and importance of mitochondrial functions in health and disease, several mitochondrial quality control processes have evolved to highly regulate the integrity, fitness, and abundance of mitochondria. These processes include mitochondrial biogenesis, mitochondrial quality control checkpoints, as well as mitochondrial degradation in the case of extended or too severe mitochondrial dysfunction [4]. The turnover and clearance of mitochondria can take place by two processes: (1) the generation and trafficking of small mitochondrial-derived vesicles (MDVs) to lysosomes upon mild mitochondrial stress; and (2) the degradation of entire damaged mitochondria through mitophagy in the most severe cases [5].

Mitophagy is a selective form of macroautophagy (hereafter referred to as autophagy) that selectively targets mitochondria to lysosomal degradation [6]. The physiological roles of mitophagy can be divided in three major branches: (1) basal mitophagy for mitochondrial maintenance; (2) programmed mitophagy necessary for different cell differentiation pathways [7]; and (3) stress-induced mitophagy in case of nutrient starvation [8], iron depletion [9], or hypoxia [10]. In addition, regarding the benefits that pathogenic bacteria could exploit from the host mitochondrial functions and homeostasis, more and more evidence recently showed that pathogens have evolved strategies to manipulate host cell mitochondria through mitophagy. Interestingly, some forms of mitophagy are reminiscent of xenophagy, another selective form of autophagy that targets intracellular pathogens for lysosomal degradation [11]. In this review, we will focus on the different mechanisms of mitophagy that have been recently identified and that can be initiated or inhibited by several bacterial pathogens able to manipulate the host cell mitophagy for survival and/or dissemination. We will however not emphasize novel modes of mitophagy such as piecemeal mitophagy involving MDVs [12] as, so far, these new mechanisms are not known to be controlled and/or manipulated by bacteria.

## 2. The diversity of mitophagy pathways

### 2.1. The generation of the autophagosome for mitophagy

**Fig 1:**
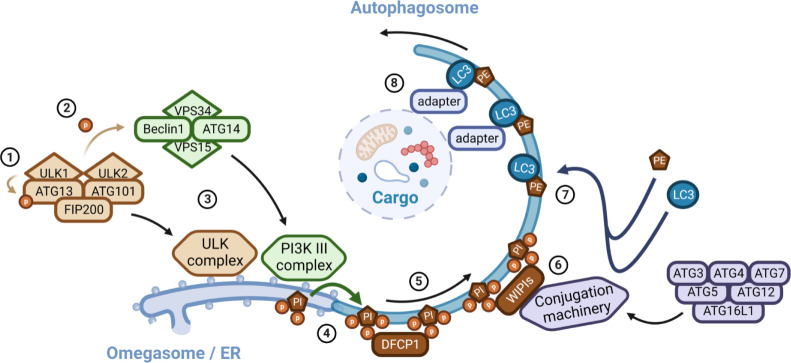
Autophagosome formation machinery. (**1**) The initiation of autophagosome formation requires the activation of the ULK complex (composed of ULK1/2 (unc-51-like kinases 1 and 2), ATG13, ATG101, and FIP200 (also known as RB1CC1)), which further activates ATG13 inducing its translocation to the ER where autophagosome formation occurs. (**2**) The ULK complex also activates and recruits (**3**) the class III PI3K lipid kinase complex (composed of VPS34, VPS15, Beclin 1, and ATG14) to the ER. (**4**) There, the PI3K III complex phosphorylates surrounding PI2P from the ER membrane, generating a PI3P-rich membrane. (**5**) The local PI3P enrichment of the ER membrane allows the recruitment of PI3P-binding proteins such as DFCP1 promoting the formation of a particular compartment termed “omegasome” from which autophagosomes are generated. (**6**) Other PI3P-binding proteins such as WIPI proteins are required for the formation of the isolation membrane of the future autophagosome, also called phagophore. WIPI proteins bind to and bring the ATG5-ATG12-ATG16L1 complex to the isolation membrane where it acts as a conjugating system, with ATG3, ATG4, and ATG7. (**7**) This machinery conjugates a PE to the LC3 type I protein converting it into LC3 type II (or LC3-PE), which is therefore incorporated in the isolation membrane, allowing its elongation and closure into an autophagosome. (**8**) LC3-PE then interacts with the targeted cargo to be degraded through specific adapters that harbour an LIR motif, allowing its sequestration inside the autophagosome, and further degradation through the lysosomal pathway. Created with BioRender.com. DFCP1, double FYVE-containing protein 1; ER, endoplasmic reticulum; LIR, LC3-interacting region; PE, phosphatidylethanolamine; PI2P, phosphatidylinositol-2-phosphate; PI3K, phosphatidylinositol-3-phosphate kinase; PI3P, phosphatidylinositol-3-phosphate; ULK, unc-51-like kinase; VPS34, vacuolar sorting protein 34; WIPI, WD repeat domain phosphoinositide-interacting.

For their degradation, mitochondria undergo several steps as it first need to be molecularly primed with several autophagy signals (also called “eat-me” signals), recognised by specific adapters or receptors, which nature depends on the type of stress mitochondria encounter. This molecular targeting allows, in a second step, the recruitment of the autophagy machinery and the engulfment of targeted mitochondria inside a double membrane vacuole, called autophagosome, which subsequently fuse with lysosomes allowing its degradation [13]. The autophagosome formation is a common feature to all selective and nonselective autophagy pathways, which is controlled by autophagy-related genes (ATGs) and is artificially divided (as it is a continuum) in four successive steps including (1) the initiation; (2) the nucleation; (3) the elongation; and (4) the sealing and maturation of the newly formed autophagosome (Fig 1) [14]. Autophagy initiation relies on the activation of the ULK (unc-51 like kinases) and class III PI3K (phosphatidylinositol-3-phosphate kinase) complexes, which are recruited at the endoplasmic reticulum (ER) to promote the formation of the phagophore [15]. The ATG12-ATG5-Atg16L1 conjugation complex is then recruited to the phagophore to allow its elongation through the incorporation of phosphatidylethanolamine (PE)-conjugated LC3, also called LC3 type II [16]. Proteins such as GATE-16 (Golgi-associated ATPase enhancer of 16 kDa) and from the GABARAP (GABA type A receptor-associated protein) family are other targets of the conjugation system that play a role in the phagophore elongation [17]. Autophagosome closure is then followed by fusion with lysosomes and degradation of its cargo [14]. While the ER appears to be a central hub for autophagosome formation, membranes from different compartments (such as recycling endosomes, the plasma membrane, the Golgi apparatus, or even mitochondria) can also gather and assemble to form the phagophore [14].

**Fig 2:**
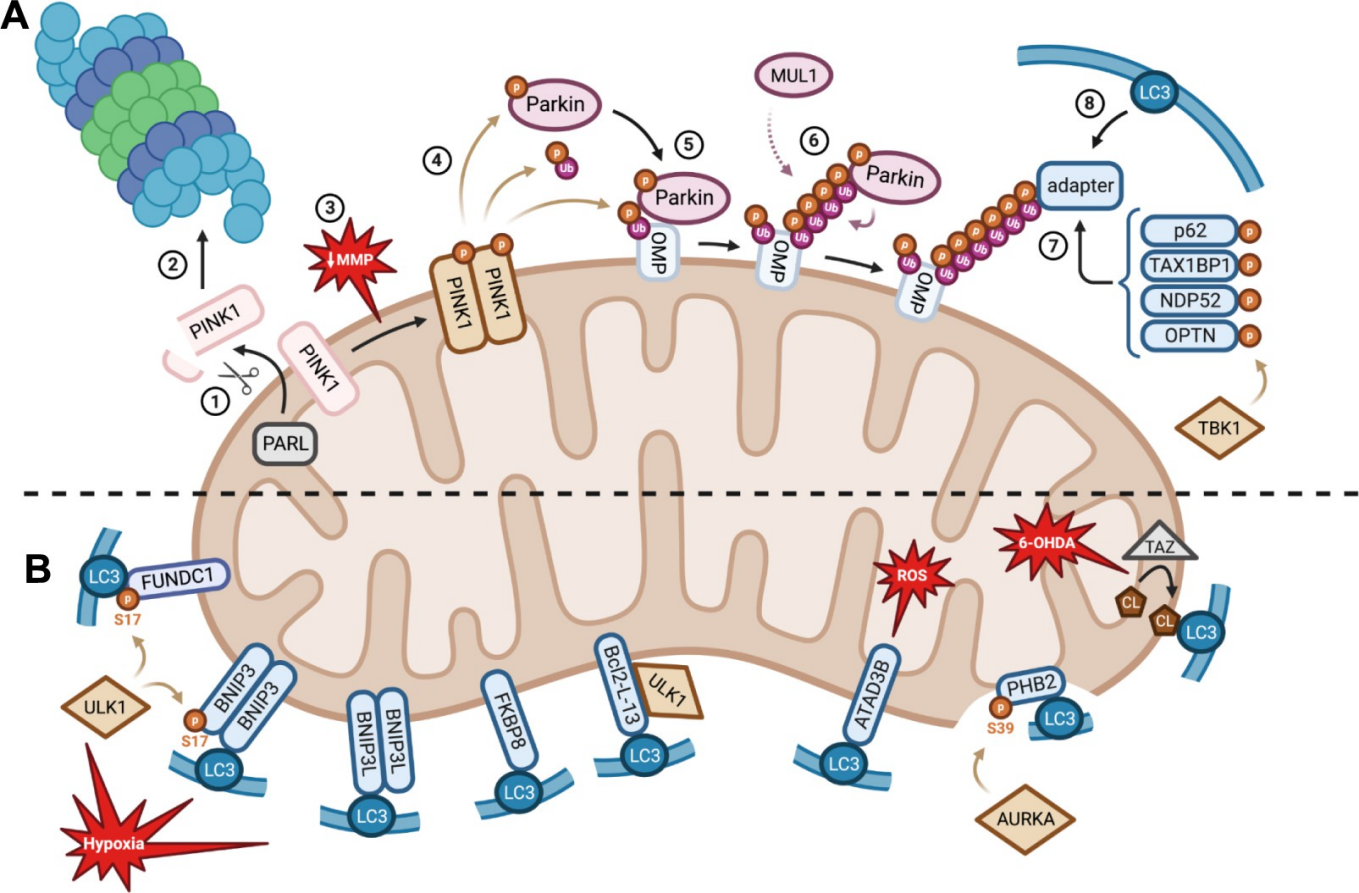
Ubiquitin-dependent and independent pathways of mitophagy. (**A**) Canonical ubiquitin-dependent mitophagy. In basal conditions, the PINK1 serine/threonine protein kinase precursor is targeted to healthy mitochondria thanks to its MTS, allowing its interaction with the TOMM complex and its import from the OMM to the IMM in an MMP-dependent way. (**1**) PINK1 then undergoes controlled proteolysis at its N-terminal part by the IMM-resident PARL protease. (**2**) Cleaved PINK1 is then released into the cytosol where it is fully degraded by the UPS. (**3**) However, upon a drop in MMP, PINK1 is no more cleaved at the IMM but is stabilised at the OMM where it accumulates as a dimeric form and activates through autophosphorylation. (**4**) PINK1 then phosphorylates ubiquitin (at serine 65) as well as Parkin (at the N-terminal ubiquitin-like domain serine 65 residue) which is found in the cytosol in an autoinhibited form. PINK1 phosphorylation of ubiquitin has been shown to be necessary for partial activation of Parkin, revealing its ubiquitin-binding domain and allowing PINK1 recognition and phosphorylation of Parkin, converting it in its fully active form. (**5**) Parkin is then recruited at the OMM through a still unclear mechanism in which ubiquitinated OMM proteins (OMP) (through the resident mitochondrial E3 ubiquitin ligase MITOL/March5) subsequently phosphorylated by PINK1 would serve as recruitment platform for activated Parkin. (**6**) Parkin recruitment at the OMM favours the nonselective polyubiquitination of OMM proteins in a positive feedforward amplification loop since PINK1-dependent polyphosphorylation of ubiquitin acts as a receptor for activated Parkin, therefore enhancing OMM protein polyubiquitination. Nonselective polyubiquitination of OMM proteins can also be performed by MUL1 independently of PINK1. (**7**) Polyubiquitin chains are then recognised by protein adapters such as p62 (also called SQSTM1), TAX1BP1, NDP52, or OPTN, previously activated by TBK1. (**8**) These adapters will finally recruit LC3-positive phagophores to form mitophagosomes. (**B**) Ubiquitin-independent or receptor-mediated mitophagy. Several LIR motif-containing receptors, which are expressed at the OMM upon different stresses, can directly interact with the LC3-positive phagophores to form mitophagosomes. FUNDC1 and BNIP3 (activated by ULK1) as well as BNIP3L and FKBP8 trigger mitophagy upon hypoxia. ATAD3B initiates mitophagy upon oxidative damage of mtDNA. PHB2 (activated by AURKA) induces mitophagy upon OMM rupture and IMM exposure to the cytoplasm. Cardiolipin is an IMM glycerophospholipid that is translocated to the OMM and triggers 6-OHDA-induced mitophagy. Bcl2-L-13 is another mitophagy receptor that requires ULK1 for proper interaction with LC3. Created with BioRender.com. FKBP8, FK506 binding protein 8; FUNDC1, FUN14 domain-containing protein 1; IMM, inner mitochondrial membrane; LIR, LC3-interacting region; MMP, mitochondrial membrane potential; MTS, mitochondria targeting signal; MUL1, mitochondrial ubiquitin ligase 1; NDP52, nuclear dot protein 52; OMM, outer mitochondrial membrane; OPTN, optineurin; PARL, presenilin-associated rhomboid-like protein; PHB2, Prohibitin 2;PINK1, phosphatase and tensin homolog-induced putative kinase 1; SQSTM1, sequestosome 1; TAX1BP1, tax 1 binding protein 1; TBK1, tank-binding kinase 1; TOMM, translocase of the outer mitochondrial membrane; UPS, ubiquitin-proteasome system.

While the autophagosome formation process is common to all autophagic responses, different types of pathways control the induction of mitophagosome formation. These pathways are classified upon their dependency or not on ubiquitin and present a panel of mitophagy-promoting receptors (Fig 2). These receptors possess an LC3-interacting region (LIR) motif allowing direct interaction with LC3-II, recruitment of the elongating phagophore, and subsequent generation of the mitophagosome (Fig 2B).

### 2.2. The ubiquitin-dependent mitophagy pathways

#### 2.2.1. The PINK1/Parkin pathway

The first described and therefore most characterised mitophagy pathway is composed of the two key proteins, PINK1 (phosphatase and tensin homolog (PTEN)-induced putative kinase 1) and Parkin, which have been identified in Parkinson’s disease, the second most common neurodegenerative disease. From the molecular point of view, the PINK1/Parkin axis is known to be induced upon several mitochondrial stresses such as a drop in MMP (mitochondrial membrane potential) as triggered by uncoupling reagents such as CCCP (carbonylcyanure m-chlorophenylhydrazone) [18], oxidative stress [19], or hypoxia [20]. The mechanisms of PINK1/Parkin-mediated mitophagy have been already widely reviewed [21] but the major regulatory processes are summarised in Fig 2A.

#### 2.2.2. The MUL1 pathway

MUL1 (mitochondrial ubiquitin ligase 1) is another E3 ubiquitin ligase that triggers ubiquitin-dependent mitophagy and has been shown to be activated in parallel with the PINK/Parkin pathway, notably during the degradation of paternal mitochondria after oocyte fertilisation [22]. In addition, MUL1 pathway activation also compensate for loss of PINK1 and/or Parkin in some models of Parkinson’s disease [23]. However, the molecular mechanisms by which MUL1 induces mitophagy are still unknown. MUL1 has also been described to inhibit PINK/Parkin mitophagy in mature neurons by reinforcing ER–mitochondria contacts and integrity through the regulation of MFN2 activity [24].

### 2.3. The ubiquitin-independent mitophagy pathways

#### 2.3.1. Receptor-mediated mitophagy

**FUNDC1:** FUNDC1 (FUN14 domain-containing protein 1) is a mitophagy receptor localised in the OMM that has been described as a major effector of mitophagy initiation upon hypoxia [10]. FUNDC1-mediated mitophagy is notably involved in cardiovascular troubles such as hypoxia in ischemia/reperfusion (IR) injuries, cardiac hypertrophy, or obesity-induced heart dysfunction [25]. Mechanistically, FUNDC1 interaction with LC3-II is regulated by several posttranslational modifications [26]. In unstressed/basal conditions, FUNDC1 is phosphorylated by CK2 (casein kinase 2) at serine 13 and by the Src kinase at tyrosine 18, which prevent interaction with LC3-II and concomitant mitophagy. However, dephosphorylation of FUNDC1-Ser13 by PGAM5 (phosphoglycerate mutase family member 5) [27] and FUNDC1-Tyr18 by NLRX1 (NOD-like receptor X1) [28] triggers mitophagy in models of hypoxia and IR injuries. In addition, upon hypoxia and mitochondrial uncoupling treatment (such as CCCP), ULK1 translocates to mitochondria to phosphorylate FUNDC1 at serine 17, which promotes LC3-II recruitment and subsequent mitophagy [29] (Fig 2B). MARCH5 (membrane-associated ring finger (C3HC4) 5, also known as MITOL (mitochondrial E3 ubiquitin ligase)) has also been shown to inhibit FUNDC1 through ubiquitination on lysine 119, leading to its proteasomal degradation [30].

**BNIP3 and BNIP3L/NIX:** BNIP3 (BCL2/adenovirus E1B 19 kDa protein-interacting protein 3) and the closely related BNIP3L (BNIP3-like, also called NIX) were first described as OMM-resident proapoptotic proteins considering their typical Bcl2 homology 3 (BH3) domain [31]. Indeed, upon apoptotic stresses, BH3 domain proteins bind and activate Bax and Bak [32]. This leads to mitochondrial outer membrane permeabilisation (MOMP) and subsequent release of proapoptotic effectors such as Smac and cytochrome c into the cytosol where they contribute to the activation of caspase-mediated cell death, or apoptosis [33]. In addition, the BH3 domain of BNIP3 and BNIP3L is also able to initiate general autophagy by releasing Beclin 1 from the antiapoptotic proteins Bcl-2 and Bcl-Xl upon hypoxia in normal and tumour cells [34]. However, BNIP3 and BNIP3L require their LIR domain to specifically trigger mitophagy [35,36]. In hypoxic conditions, the expression of BNIP3 and BNIP3L is induced through the stabilisation and activation of HIF-1α (hypoxia inducible factor 1 α), which tightly regulates both genes expression [37]. To fulfil their role as mitophagy receptors, BNIP3 and BNIP3L require posttranslational modifications. As ULK1 promotes BNIP3 stabilisation and activation through phosphorylation at serine 17 [38], both BNIP3 and/or BNIP3L homodimerisation is essential for proper mitophagy initiation at the OMM [39] (Fig 2B). Regarding BNIP3, it is the phosphorylation status of serine 17 and 24 in the LIR motif that determines its role in prosurvival mitophagy or apoptosis [40].

Although BNIP3 and BNIP3L mitophagy pathways were first thought to be distinct of the ubiquitin-dependent PINK1/Parkin pathway, recent studies demonstrate a tight regulation between these two mechanisms. Indeed, BNIP3 also suppresses the proteolytic cleavage and inactivation of PINK1 to promote ubiquitin-dependent mitophagy [41]. In addition, up-regulation of BNIP3L through iron depletion has been shown to promote a strong activation of the PINK1/Parkin mitophagy to induce cell death in a model of wild-type p53 colon carcinoma [42]. In the case of Parkin deficiency, BNIP3L is even able to compensate the lost role of Parkin, for instance, in a model of cadmium-induced mitophagy in HeLa cells [43].

**FKBP8:** In basal conditions, FKBP8 (FK506 binding protein 8, also called FKBP38) is anchored to the OMM where it localises and stabilises the antiapoptotic factors Bcl-2 and Bcl-Xl to the mitochondria to suppress apoptosis [44]. Upon nutrient starvation, FKBP8 delocalises from the mitochondria and act as an endogenous inhibitor of mTOR (mechanistic target of rapamycin, the central regulator of cell growth, autophagy, and metabolism) whose inhibitory activity is suppressed by Rheb (a RAS-like small GTPase) [45], as well as upon interaction with PHB1 (prohibitin1), an inner mitochondrial membrane (IMM) protein that sequestrates FKBP8 at the mitochondria [46]. More recently, a LIR motif have been identified in FKBP8 responsible for mitophagy upon hypoxia and CCCP treatment in a PINK1/Parkin-independent manner [47] (Fig 2B). However, FKBP8 is not degraded during mitophagy as it is released from the mitochondria and localises at the ER during mitophagy [47,48]. The functional role of the delocalisation of FKBP8 is not clear yet but one hypothesis might be that FKBP8 translocation to the ER would suppress apoptosis during mitophagy [48].

**BCL2-L13:** Bcl2-L-13 (Bcl2-like protein 13) is an OMM-anchored protein that corresponds to the mammalian ortholog protein of the yeast Atg32 mitophagy receptor [49]. Bcl2-L-13 induces mitochondrial fragmentation and mitophagy during nutrient starvation in a PINK1/Parkin-independent manner. The ULK1 initiation complex has been shown to directly bind to Bcl2-L13 upon nutrient starvation to allow LC3 recruitment and autophagosome formation around damaged mitochondria [50] (Fig 2B).

**PHB2:** Upon OMM rupture through proteasomal degradation of OMM proteins, IMM exposure to the cytosol can be a key signal for mitophagy to clear damaged mitochondria. In this case, PHB2 (Prohibitin 2), an IMM integral protein found as heterodimers with PHB1, is a mitophagy receptor that interacts with LC3-II when exposed to the cytosol [51]. The molecular mechanisms of PHB2 activation upon mitochondrial damage involve AURKA (Aurore Kinase A), which interacts with PHB2 and activates it through phosphorylation on serine 39 [52]. This activation is required for the subsequent interaction with LC3-II and formation of the mitophagosome (Fig 2B).

**ATAD3B:** ATAD3B (ATPase Family AAA Domain Containing 3B) is a protein that regulates the stabilisation of large mtDNA-proteins complexes called nucleoids [53] and acts as a mitophagy receptor that clears mitochondria upon mtDNA oxidative damage [54]. ATAD3B is a transmembrane integral protein that constitutively interacts with ATAD3A, but upon oxidative stress and damaged mtDNA accumulation, the C-terminal region of ATAD3B translocates from the mitochondrial intermembrane space to the OMM, exposing its LIR motif towards the cytoplasm allowing the elimination of oxidative damaged mitochondria [54].

#### 2.3.2. Cardiolipin-mediated mitophagy

In eukaryotic cells, the cardiolipin is a mitochondria-exclusive glycerophospholipid found in the IMM and promotes, when aggregated, the IMM curvature due to its typical shape defined by a double glycerophosphate backbone and four fatty acyl chains [55]. While its role in mitochondrial membrane structure, protein import, and bioenergetics is well known, cardiolipin can also serve as a danger signal upon mitochondrial damage. Indeed, in a model of neurons exposed to neurotoxins such as 6-OHDA (6-hydroxydopamine), cardiolipin is translocated from the IMM to the OMM and directly interacts with LC3-II for mitophagosome formation [56]. Several proteins have been identified to help cardiolipin redistribution to the OMM such as the phospholipid transacylase TAZ [57] and the phospholipid scramblase 3 [56]. Cardiolipin-mediated mitophagy has been suggested to prevent cardiolipin oxidation, which would subsequently lead to mitochondrial membrane damage, cytochrome c release, and apoptosis [56].

## 3. The battle between bacterial pathogens and host mitophagy: Rather prosurvival or antibacterial?

### 3.1. Intracellular pathogens

#### 3.1.1. Pseudomonas aeruginosa

*P*. *aeruginosa* is a motile gram-negative rod-shaped opportunistic extracellular pathogen and represents a major cause of life-threating nosocomial infections including hospital-acquired pneumonia or chronic obstructive pulmonary disease (COPD) [58]. In lung diseases, acute *P*. *aeruginosa* infections can eventually evolve into a chronic status through to the development of a variety of virulence effectors allowing pathological adaptation and colonisation of the epithelium into a mucoid biofilm [59]. These virulence factors include the following: (1) lipopolysaccharide (LPS) and porins; (2) the flagellum and pili; (3) the type 3 protein secretion system (T3SS) responsible for direct injection of toxic effectors inside of the host cell to trigger cell death, immune system alteration and bacterial dissemination; (4) the exotoxin A, which inhibits host protein synthesis and promotes apoptosis; and (5) siderophores such as pyoverdine and pyochelin, which scavenge iron form host cell proteins to the bacterium [59].

**Fig 3:**
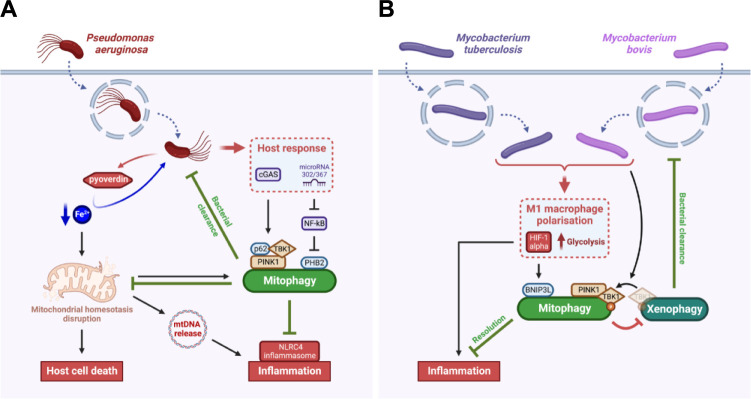
Intracellular pathogens have evolved strategies to escape the defensive mitophagy responses induced by the host. (**A**) *Pseudomonas aeruginosa* induces the cell death of mammalian cells and an NLRC4 inflammatory response through a pyoverdine-dependent iron starvation response and subsequent mitochondrial damage. While host cells activate a defence mitophagy response through PINK1/p62- and PHB2-dependent axis correlated with increased bacterial clearance, this mitophagy also attenuates the host cell death and inflammation, which preserve *P*. *aeruginosa* replication niche. (**B**) *Mycobacterium* spp. can trigger in macrophages either a M1 or a M2 phenotype polarisation. In the case of M1 polarisation, the infected macrophage triggers a pro-inflammatory phenotype through the activation of HIF-1α and up-regulation of glycolysis. However, the BNIP3L-mediated mitophagy induced by *M*. *tuberculosis* and *M*. *bovis* through HIF-1α attenuates the M1 inflammatory response. In addition, *M*. *bovis* triggers the translocation of the phosphorylated form of TBK1 from the xenophagy machinery to the mitophagy machinery, therefore inhibiting xenophagy-mediated bacterial clearance. In both models, the pathogen has evolved mechanisms to escape the defensive mitophagy response triggered by the host. Created with BioRender.com. HIF-1α, hypoxia inducible factor 1 α; NLRC4, NOD-like receptor family caspase recruitment domain containing 4; PINK1, phosphatase and tensin homolog-induced putative kinase 1; TBK1, tank-binding kinase 1.

When *P*. *aeruginosa* is found inside the host cell, iron extraction and transport by the pyoverdine leads to mitochondrial homeostasis disruption and subsequent host cell death [60]. As a mitochondrial surveillance response, the host cell induces a PINK1-Beclin1-dependent-mitophagy to eliminate damaged mitochondria accumulating upon *P*. *aeruginosa* infection [61] (Fig 3A). In addition, the PINK1-Beclin1-mitophagy pathway protects the host against *P*. *aeruginosa* as it helps the clearance of the pathogen in a similar way as xenophagy, that was already reported for that bacteria [61]. In addition, upon *P*. *aeruginosa* infection, other mitophagy mechanisms are also induced by the host itself to limit inflammation. Indeed, mitophagy triggered by *P*. *aeruginosa*-induced mtDNA release attenuates the activation of the NLRC4 (NOD-like receptor family caspase recruitment domain containing 4) inflammasome [62]. Moreover, the microRNA-302/367 up-regulated upon *P*. *aeruginosa* infection cluster activates a PHB2-dependent mitophagy pathway to increase *P*. *aeruginosa* clearance and limit inflammation through a negative regulation of NF-kB [63]. In addition, the cGAS (cyclic GMP-AMP synthase) pathway is essential for host defence against *P*. *aeruginosa* since it induces a PINK1-TBK1-p62-dependent mitophagy response associated with bacterial elimination and reduction of inflammation [64].

#### 3.1.2. Mycobacterium tuberculosis

*M*. *tuberculosis* is a rod-shaped intracellular pathogen harbouring one peculiar cell membrane with arabinogalactan associated to peptidoglycan and covered by a thick layer of mycolic acids [65]. *M*. *tuberculosis* is the etiologic agent of tuberculosis, the leading cause of death from bacterial infectious disease worldwide [66]. *M*. *tuberculosis* is a particularly adaptable pathogen that coevolved with its human host since most infections are asymptomatic (latent tuberculosis) until the host immune response is compromised. Indeed, *M*. *tuberculosis* resides in the lung alveoli where it manipulates the host immunity leading to the recruitment and aggregation of diverse types of immune cells in the infection site [67]. These nodules, called granulomas, are extremely heterogenous and dynamic within individuals making *M*. *tuberculosis* difficult to eradicate. Moreover, the yearly rise of MDR strains supports the need for novel therapeutic approaches.

Recently, a study highlighted thiopeptides as emerging clinical antibiotic candidates for *M*. *tuberculosis* elimination through their direct antibacterial activity and induction of host mitophagy [68]. This study relies on previous research showing that mitophagy is triggered in macrophages upon *M*. *tuberculosis* infection [69] (Fig 3B). More precisely, *M*. *tuberculosis* and *M*. *bovis* infections are known to promote M1 macrophage polarisation with a pro-inflammatory phenotype among other polarisation programs [70]. In the case of M1 polarisation, the up-regulation of glycolysis and the induction of a BNIP3L-mediated mitophagy are correlated with a better resolution of the infection [71]. Henceforth, targeting host mitophagy with new therapies such as thiopeptides sounds promising for *M*. *tuberculosis* treatment. However, whether mitophagy is directly linked to bacterial elimination is not clear. Indeed, a recent study suggests that *M*. *bovis*-induced mitophagy is rather beneficial for the bacteria as mitophagy suppresses host xenophagy for intracellular survival [72]. The PINK1-dependent mitophagy induced by *M*. *bovis* requires phosphorylated TBK1 (p-TBK1), which is needed for xenophagy of the bacteria [73]. The competitive utilisation of p-TBK1 between mitophagy and xenophagy helps thus *M*. *bovis* to escape from degradation through xenophagy [72] (Fig 3B). The differences between both *M*. *tuberculosis* and *M*. *bovis*-mediated mitophagy responses require further research to clarify whether mitophagy is beneficial for the bacteria or represents a defensive response from the host, or both.

#### 3.1.3. Listeria monocytogenes

*L*. *monocytogenes* is a gram-positive rod-shaped facultative intracellular pathogen that causes listeriosis, a foodborne associated infection that can affect animals as well as humans [74]. The virulence of *L*. *monocytogenes* relies on its ability to cross the intestine barrier and reach the liver and spleen. In immunocompromised individuals, *L*. *monocytogenes* crosses the blood–brain barrier and the fetoplacental barrier leading to fatal meningitis or abortion [75]. At the cellular level, *L*. *monocytogenes* escapes from the endolysosomal pathway through permeabilisation of its vacuole, then it polymerises actin at one pole to acquire motility and spread to a neighbouring cell [76].

**Fig 4:**
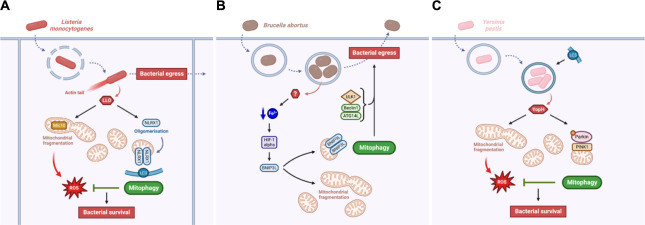
Intracellular pathogens actively induce mitophagy for bacterial survival and egress. (**A**) *Listeria monocytogenes* virulence relies on LLO. LLO induces Mic10-dependent mitochondrial fragmentation correlated with an increase in mtROS production, which might be detrimental for the pathogen. However, LLO also triggers NLRX1-mediated mitophagy, which promotes the elimination of damage mitochondria and therefore inhibit mtROS accumulation for bacterial survival and egress. (**B**) *Yersinia pestis* manipulates its host cell mitochondria in a similar way as *L*. *monocytogenes* does. *Y*. *pestis* virulence relies on the YopH effector, which induces mitochondrial fragmentation, mtROS release, and PINK1/Parkin-mediated mitophagy. As for *L*. *monocytogenes*, this mitophagy response limits mtROS accumulation and enables bacterial survival. (**C**) *Brucella abortus* induces mitochondrial fragmentation and BNIP3-mediated mitophagy through a still unknown effector. This mitophagy response promote bacterial egress in addition with other autophagy actors such as ULK1, Beclin1, and ATG14L. In these three models, the pathogen manipulates mitochondrial morphology and degradation either to avoid oxidative stress and favour its own survival and/or to enable its dissemination in the host. Created with BioRender.com. LLO, listeriolysin O; mtROS, mitochondrial reactive oxygen species; NLRX1, NOD-like receptor X1; PINK1, phosphatase and tensin homolog-induced putative kinase 1.

*L*. *monocytogenes* is also able to manipulate the host mitochondria, notably by triggering mitochondrial fragmentation mediated by its virulence effector listeriolysin O (LLO) [77] (Fig 4A). LLO up-regulates the expression of the MICOS (mitochondrial contact site and cristae organizing system) complex subunit Mic10, which is important for IMM ultrastructure and is necessary for *L*. *monocytogenes*-induced mitochondrial fragmentation [77,78]. However, no clear function of mitochondrial fragmentation was established for *L*. *monocytogenes* intracellular lifecycle. Mitochondrial fission being one hallmark of mitophagy, *L*. *monocytogenes* also triggers mitophagy by a recently identified mitophagy receptor, the NOD-like receptor NLRX1 [79]. Mechanistically, LLO promotes the oligomerisation of NLRX1, which favours the binding of its LIR motif to LC3 and subsequent mitophagy that limits mtROS production generated from the *L*. *monocytogenes*-induced mitochondrial fragmentation. Therefore, the NLRX1-mediated mitophagy triggered by LLO is beneficial for *L*. *monocytogenes* survival in the host cell as it avoids bacteria to be killed by oxidative stress generated by damaged mitochondria [79]. It could be quite counterintuitive to think that *L*. *monocytogenes* needs to induce mitochondrial fragmentation through LLO for its lifecycle, since it generates bacterial-killing mtROS release. However, *L*. *monocytogenes* could actively trigger mitophagy (through LLO) to modulate or compensate for the deleterious accumulation of mtROS caused by the presence of the bacteria.

#### 3.1.4. Yersinia pestis

*Y*. *pestis* is a gram-negative facultative intracellular coccobacilli that is the causative agent of the plague inoculated by flea vectors (for a historical review, read [80]). The typical lymph node infections induced by *Y*. *pestis* are characterised by its extracellular lifestyle orchestrated by the *Yersinia* outer proteins (Yops), which are injected inside host cells through a T3SS [81]. However, *Y*. *pestis* virulence also occurs through intracellular trafficking and persistence inside the host. Following phagocytosis, *Y*. *pestis* is able to target several Rab GTPases (Rab4a, Rab1b, and Rab11b) to the phagosome to inhibit its acidification and disrupt host cell recycling, therefore leading to bacterial replication inside a nonacidic autophagosome [82].

More recently, another prosurvival mechanism deployed by *Y*. *pestis* has been identified and is linked to manipulation of host mitochondria [83] (Fig 4B). Similarly to *L*. *monocytogenes*, *Y*. *pestis* infection leads to mitochondrial fragmentation causing an increase in mtROS release, which display antibacterial properties. However, requiring the YopH effector, *Y*. *pestis* triggers a PINK1/Parkin-dependent mitophagy response to clear dysfunctional mitochondria induced by the infection, limiting the accumulation of mtROS and allowing bacterial persistence [83]. *Y*. *pestis* and *L*. *monocytogenes* are thus two pathogens for which mitophagy is activated by a virulence effector to clear the host cell from pathogen-induced damaged mitochondria and therefore preserve the replicative niche.

#### 3.1.5. Brucella abortus

*B*. *abortus* is a gram-negative facultative intracellular coccobacilli that causes brucellosis, a worldwide zoonosis affecting domestic animals and humans [84]. Animal brucellosis leads to abortion and infertility, therefore generating significant economic losses [85]. As an intracellular pathogen, *B*. *abortus* virulence relies on the expression of a type 4 secretion system (T4SS) named VirB, which allows effector injection inside the host cell [86]. These effectors mainly manipulates the host cell secretory pathway for *B*. *abortus* to reach its replicative niche inside the ER, called replicative *Brucella*-containing vacuoles (rBCVs) [87]. Eventually, *B*. *abortus* subverts the host cell autophagy initiation machinery (including ULK1, Beclin1, and ATG14L) to help the formation of autophagic-like BCVs (aBCVs), which are necessary for bacterial egress from the host cell [88].

Recent studies showed that *B*. *abortus* also interacts with the host mitochondria, inducing severe mitochondrial network fragmentation during the late steps of cellular infection [89] (Fig 4C). In addition, *B*. *abortus* simultaneously triggers mitophagy in an iron-HIF-1α-BNIP3L-dependent manner [90]. As for the actors of autophagy initiation, this mitophagy response also plays a role in *B*. *abortus* exit of host cells through an alteration of aBCVs formation [90]. This could mean that *B*. *abortus*-induced mitophagy would favour the spreading of the bacteria from cell to cell, and even towards other tissues. However, as no *B*. *abortus* virulence effector has been identified yet as a driver of mitophagy induction, further research would be needed to decipher whether BNIP3L-mediated mitophagy is an active response mediated by *B*. *abortus* or not. However, in the case of *B*. *abortus*, the induction of mitophagy is linked to bacterial dissemination, and not to bacterial replication. This highlights a novel process by which mitophagy could be beneficial for the persistence of some pathogens.

### 3.2. Extracellular pathogens

#### 3.2.1. Helicobacter pylori

*H*. *pylori* is a gram-negative spiral multiflagellar extracellular pathogen that colonises the epithelium of the human stomach [91]. *H*. *pylori* infection leads to the development of several gastric diseases such as chronic gastritis, ulcers, mucosa-associated lymphoid tissue lymphoma, and gastric cancers. One of the main and most studied toxin of *H*. *pylori* is VacA (vacuolating cytotoxin A) [92]. VacA is a multifunctional toxin that can (1) oligomerise to form anion selective channels inserted in late endosomes inducing vacuolisation of the host epithelial cells impairing its protein secretion pathway; (2) manipulate the host cell death mechanisms; (3) disrupt the epithelial cell–cell junctions; and (4) interfere with the function of most of the immune cells.

**Fig 5:**
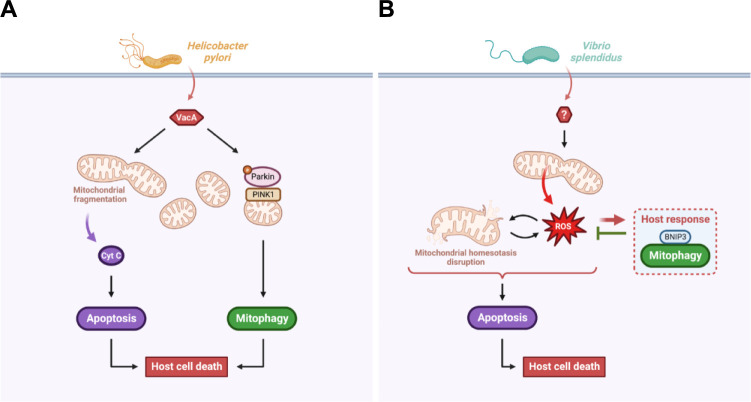
Extracellular pathogens are also able to induce mitophagy to regulate the host cell death. (**A**) *Helicobacter pylori* virulence relies on the VacA effector, which induces mitochondrial-mediated apoptosis, as well as PINK1/Parkin-mediated mitophagy, both leading to the host cell death. (**B**) *Vibrio splendidus* triggers an increase in mtROS accumulation causing mitochondrial damage and exacerbated accumulation of mtROS, which leads to host cell death through apoptosis. Mitochondrial damage induces in the host a defensive BNIP3-mediated mitophagy response, which eliminates damage mitochondria and limits host cell death. Created with BioRender.com. mtROS, mitochondrial reactive oxygen species; PINK1, phosphatase and tensin homolog-induced putative kinase 1; VacA, vacuolating cytotoxin A.

VacA manipulates several host cell signaling pathways to induce its death either through apoptosis and/or mitophagy (Fig 5A). Indeed, VacA is targeted to the OMM where it initiates mitochondrial network fission, MMP disruption, cytochrome c release, and subsequent host apoptotic cell death [93]. In addition, VacA is also targeted to the IMM (through the TOM complexes) to further disturb the MMP and trigger a PINK1/Parkin-dependent mitophagy cell death [94]. *H*. *pylori*-induced mitophagy might also be initiated through the translocation of STAT3 (Signal Transducer and Activator of Transcription 3) phosphorylated on the serine 727 to the mitochondria [95]. Gastric epithelial cell death induced by *H*. *pylori*-mediated apoptosis and/or mitophagy eventually leads to alteration of the gastric mucosa contributing to gastric disease for the host [91], without providing any apparent benefits for the pathogen. However, compromising the integrity of the gastric epithelial barrier is beneficial for *H*. *pylori* survival. Indeed, VacA-dependent gastric epithelial cell death is essential for nutrient release required for *H*. *pylori* growth in the gastric environment [96]. *H*. *pylori* thus represents a model of extracellular pathogen able to manipulate the host mitophagy for its own survival.

#### 3.2.2. Vibrio splendidus

*V*. *splendidus* is a widespread gram-negative rhabdoid extracellular pathogen for aquatic animals including shellfish (such as the oyster *Crassostrea gigas*), turbot fish (*Scophthalmus maximus*), or commercially important species of sea cucumber in Chinese aquaculture (*Apostichopus japonicus*) [97]. Several virulence factors of *V*. *splendidus* have been identified during *A*. *japonicus* infection. They include quorum sensing, extracellular metalloproteases, siderophores, as well as the hemolysin 4-hydroxyphenylpyruvate dioxygenase (4-hppD), which elicit a strong skin ulceration syndrome causing great economic losses in the *A*. *japonicus* aquaculture [98].

However, recent studies have shown that the host can engage prosurvival mitophagy-dependent programs to counteract the infection (Fig 5B). Upon *V*. *splendidus* infection of *A*. *japonicus* coelomocytes, MMP alterations are observed and lead to a massive load of mtROS, which sustains mitochondrial damage and the host cell death by apoptosis [99]. In response, the host develops a ROS-mediated BNIP3-dependent mitophagy response to eliminate mitochondrial damage and promote host cell survival. Moreover, the oyster host *C*. *gigas* also triggers mitophagy during *V*. *splendidus* infection, highlighting, for the first time, the existence of mitophagy in mollusks [100]. In opposition to *H*. *pylori*, *V*. *splendidus* represents a model of extracellular pathogen for which the host mitophagy is a defensive response, not sufficient, however, to completely eradicate the pathogen and resolve the infection.

## 4. Conclusions

Mitochondria are complex organelles that acquired through evolution machineries to cope with and eliminate bacterial pathogens that can infect eukaryotic host cells. These mechanisms notably include the regulation of many cellular responses such as mtROS production, regulation of apoptosis, and an important role in innate immunity. Yet several bacterial pathogens, whether they display an intracellular or extracellular lifestyle, evolved as well, being able to manipulate the biology of mitochondria to their advantage for survival purposes. One of the major means would be to drive the host cell to induce degradation of damaged mitochondria that can be deleterious for the pathogen.

Mitochondrial clearance is mainly mediated by mitophagy, a widely diversified process for which a plethora of mechanisms and molecular actors have been identified (Fig 2). Interestingly, there is an overlap between the molecular actors regulating mitophagy and xenophagy, including the ubiquitin-binding receptors NDP52, p62, OPTN, as well as p-TBK1, which are involved as an equilibrium in both pathways [101]. As mentioned in the introduction, xenophagy selectively targets pathogens for degradation through recognition and ubiquitination of specific surface components, a mechanism that is highly reminiscent to the clearance of damaged ubiquitinated mitochondria. The common link in eliminating mitochondria and bacteria could potentially be explained by the ancestor similarities of these two entities. This common trait is, for instance, subverted by *M*. *tuberculosis*, which induces mitophagy to impair xenophagy. Amplification and relocalisation of molecular actors such as p-TBK1 from the xenophagy machinery to the mitophagy machinery benefit to pathogen survival and dissemination. In addition, like xenophagy, mitophagy is also described to help bacterial clearance upon *P*. *aeruginosa* (Fig 3A). However, future research would still be needed to better understand how mitophagy can mediate *P*. *aeruginosa* clearance.

Among others, an important question remains to be addressed: Is host cell mitophagy only manipulated by invading bacteria for their own survival? Or does the host cell induce mitophagy by itself as a defence response against the pathogen? The literature does not give a complete and firm answer to these questions, which should rather be considered as two extreme views with the possibility of an intermediate situation. Indeed, depending on the pathogen of interest, mitophagy can be at the same time induced by the host as a defence response (Fig 3) or by the pathogen for its own survival (Fig 4). Nonetheless, no cases of successful defensive mitophagy response triggered by the host against the invading pathogen was reported yet. Most pathogens trigger mitophagy, instead of inhibiting it, as a protective response to limit accumulation of damaged mitochondria and inflammation, as well as to preserve the bacterial replicative niche and prevent pathogen clearance by the host cell. Indeed, the induction of mitophagy protects against accumulation of mitochondrial damages such as mtDNA release (in the case of *P*. *aeruginosa*), or mtROS-derived oxidative stress (as reported for *L*. *monocytogenes*, *Y*. *pestis*, and *V*. *splendidus*), which could lead to the activation of the inflammasome, the maturation of pro-inflammatory IL-1β and IL-18 cytokines, and subsequent elimination of the infection [102]. However, the effect of pathogen-driven mitophagy on the initiation of specific pro-inflammatory programs, such as the interferon γ (IFNγ) response, and subsequent effect on neighbouring cells is not well known, nor abundantly studied, in the context of bacterial infections. Interesting hypotheses might however be borrowed from virology studies. Indeed, viruses such as the coxsackievirus B3, the measles virus, and the SARS-coronavirus are known to disrupt the mitochondria antiviral signalling (MAVS) proteins and subsequent IFNγ signalling through mitophagy [103]. Since mitophagy induction can limit inflammasome activation [102], further research is still needed to decipher what is the impact of the induction of mitophagy on the behaviour of neighbouring immune and nonimmune cells, especially in the context of *P*. *aeruginosa* and *L*. *monocytogenes* infection. In addition, pathogen-induced mitophagy is also related to bacterial fitness and dissemination as it helps bacterial egress and infection of neighbouring cells (in the case of *L*. *monocytogenes* and *B*. *abortus*) (Fig 4). However, the molecular mechanisms linking mitophagy to bacterial egress still need to be discovered.

When mitophagy appears to be beneficial for the pathogen survival, virulence effectors might be responsible for actively manipulating the initiation mechanisms of the host mitophagy. Several bacterial effectors have already been identified as such during cellular infection by *L*. *monocytogenes* (LLO), *Y*. *pestis* (YopH), and *H*. *pylori* (VacA). Further experimental strategies should also be considered to identify putative virulence effectors from *M*. *bovis* and *B*. *abortus*, which could be required for their active manipulation of p-TBK1 and BNIP3L, respectively.

In conclusion, even if mitochondria and bacteria could be evolutionary considered as two relatives, there is a real struggle between them to determine survival outcome during infection. Further research in the relatively recent field of pathogen-induced mitophagy would be of great interest to better understand these mechanisms at the molecular level and potentially identify new molecular actors that could pinpoint new therapeutic approaches to fight the growing threat of multidrug resistance pathogens.
